# Metazoan parasite communities: support for the biological invasion of *Barbus barbus* and its hybridization with the endemic *Barbus meridionalis*

**DOI:** 10.1186/s13071-016-1867-9

**Published:** 2016-11-17

**Authors:** L. Gettová, A. Gilles, A. Šimková

**Affiliations:** 1 Department of Botany and Zoology, Faculty of Science, Masaryk University, Kotlářská 2, 61137 Brno, Czech Republic; 2 Aix-Marseille Université, IMBE, UMR CNRS 7263, Evolution Génome Environnement, Case 36, 3 Place Victor Hugo, 13331 Marseille Cedex 3, France

**Keywords:** Cyprinid fish, Biological invasion, Hybridization, Metazoan parasite communities

## Abstract

**Background:**

Recently, human intervention enabled the introduction of *Barbus barbus* from the Rhône River basin into the *Barbus meridionalis* habitats of the Argens River. After an introduction event, parasite loss and lower infection can be expected in non-native hosts in contrast to native species. Still, native species might be endangered by hybridization with the incomer and the introduction of novel parasite species. In our study, we aimed to examine metazoan parasite communities in *Barbus* spp. populations in France, with a special emphasis on the potential threat posed by the introduction of novel parasite species by invasive *B. barbus* to local *B. meridionalis.*

**Methods:**

Metazoan parasite communities were examined in *B. barbus*, *B. meridionalis* and their hybrids in three river basins in France. Microsatellites were used for the species identification of individual fish. Parasite abundance, prevalence, and species richness were compared. Effects of different factors on parasite infection levels and species richness were tested using GLM.

**Results:**

Metazoan parasites followed the expansion range of *B. barbus* and confirmed its introduction into the Argens River. Here, the significantly lower parasite number and lower levels of infection found in *B. barbus* in contrast to *B. barbus* from the Rhône River supports the enemy release hypothesis. *Barbus barbus* × *B. meridionalis* hybridization in the Argens River basin was confirmed using both microsatellites and metazoan parasites, as hybrids were infected by parasites of both parental taxa. Trend towards higher parasite diversity in hybrids when compared to parental taxa, and similarity between parasite communities from the *Barbus* hybrid zone suggest that hybrids might represent “bridges” for parasite infection between *B. barbus* and *B. meridionalis*. Risk of parasite transmission from less parasitized *B. barbus* to more parasitized *B. meridionalis* indicated from our study in the Argens River might be enhanced in time as higher infection levels in *B. barbus* from the Rhône River were revealed. Hybrid susceptibility to metazoan parasites varied among the populations and is probably driven by host-parasite interactions and environmental forces.

**Conclusions:**

Scientific attention should be paid to the threatened status of the endemic *B. meridionalis*, which is endangered by hybridization with the invasive *B. barbus*, i.e. by genetic introgression and parasite transmission.

**Electronic supplementary material:**

The online version of this article (doi:10.1186/s13071-016-1867-9) contains supplementary material, which is available to authorized users.

## Background

Concerns over the ecological implications of the introduction of an alien species into new environments are increasing. From the conservation point of view, there is elevated apprehension if the native species represents an endemic or endangered species, which is often characterized by small population sizes, fragmented distribution and low genetic variability (e.g. [1, 2]). A new incomer may, therefore, represent a serious problem for the resident if it predates the indigenous species, exploits the same resources, or alters its native habitat [3–6]. Following the introduction event, many native species are endangered through hybridization with the closely related alien species [7–9]. At the same time, the new invader serves as a source of novel parasites to which the local species may display a different degree of susceptibility [10, 11]. Consequently, exposing susceptible local hosts to new parasite species carried by introduced individuals may result in accelerated mortality in native populations. For instance, the parasitic nematode *Anguillicoloides crassus* was imported to Europe probably as a result of the introduction of the Japanese eel *Anguilla japonica* and was, subsequently, disseminated in the populations of the European eel *Anguilla anguilla* (reviewed in [12]). While *A. crassus* is not highly pathogenic in the Japanese eel probably due to low-intensity infection rates [13], high infection and more serious pathology connected with high mortalities may be detected in wild European eels [14–17].

Populations of invaders established in new habitats typically exhibit fewer parasite species, and a smaller number of host individuals are parasitized (i.e. there is a lower prevalence of infection) when compared to the source populations [18]. This could be the result of the introduction of a restricted number of individuals carrying only a proportion of the original parasite fauna, new and unsuitable environmental conditions for parasites, and the absence or low abundance of suitable hosts required for the parasite life-cycles [19, 20]. Such a release from co-evolved parasites may therefore provide an advantage for the performance of a novel host species in new habitats [21]. However, this advantage is often of a temporal nature and parasite species richness and prevalence rates can return to original levels or even be multiplied in a short time by transmission from the local hosts [22]. Co-evolutionary relationships which evolve between hosts and their parasites during their co-existence may, therefore, be steered by ecological forces [23]. Hybridization might even alter the composition of metazoan parasite communities of the two interacting host species, since hybrid individuals are often vulnerable to parasites infecting both parental species [24, 25] and may, therefore, represent “bridges” for parasite infection [26].

In France, two congeneric *Barbus* species co-exist in several rivers in the Mediterranean basin. The low level of mitochondrial DNA and allozyme variability indicates recent colonization of the French rivers by the common barbel *Barbus barbus* after the last glaciation [27, 28], where the Mediterranean barbel *Barbus meridionalis* was already present, probably from the Miocene [27]. Nowadays, the widely distributed European species *B. barbus* has been found in almost all French river basins and prefers medium-sized to large rivers. By contrast, the occurrence of endemic *B. meridionalis* is restricted to the Languedoc-Roussillon, Rhône-Alpes, and Provence-Alpes-Côte d’Azur regions in France [27], where it inhabits mainly upper and middle streams of mountain rivers, probably as a result of competition with *B. barbus* [29]. Nevertheless, hybridization between these two species has previously been reported from the Hérault, Garonne, Orb and Rhône river basins [30]. Moreover, an irrigation canal which supplies the Var department with water collected from the River Durance (Rhône River basin) enabled the very recent immigration of *B. barbus* individuals into the habitats of the native *B. meridionalis* approximately 30 years ago [31, 32]. While Kiener et al. [33] in 1981 rejected the presence of *B. barbus* in the River Argens, hybridization between *B. barbus* and *B. meridionalis* is already occurring in this river [34].

In our study, we aimed to examine the composition of metazoan parasite communities of *B. barbus* and *B. meridionalis* in (i) allopatric areas, (ii) sympatric areas of late origin of the Rhône River basin, and (iii) sympatric areas of recent origin of the Argens River basin. In the Argens River basin, we further intended to confront the metazoan parasite communities of parental taxa with those found in hybrids which resulted from the biological invasion of *B. barbus* into this watershed. The metazoan parasite abundance, prevalence and species richness in *Barbus* populations collected from the three areas were analyzed. We focused on the possible threat posed by the introduction of the widely distributed *B. barbus* to native and endemic *B. meridionalis* with respect to the transmission of non-native parasite species and the role of hybrids in facilitating parasite transmission between parental taxa.

## Methods

### Sample collection

**Fig. 1:**
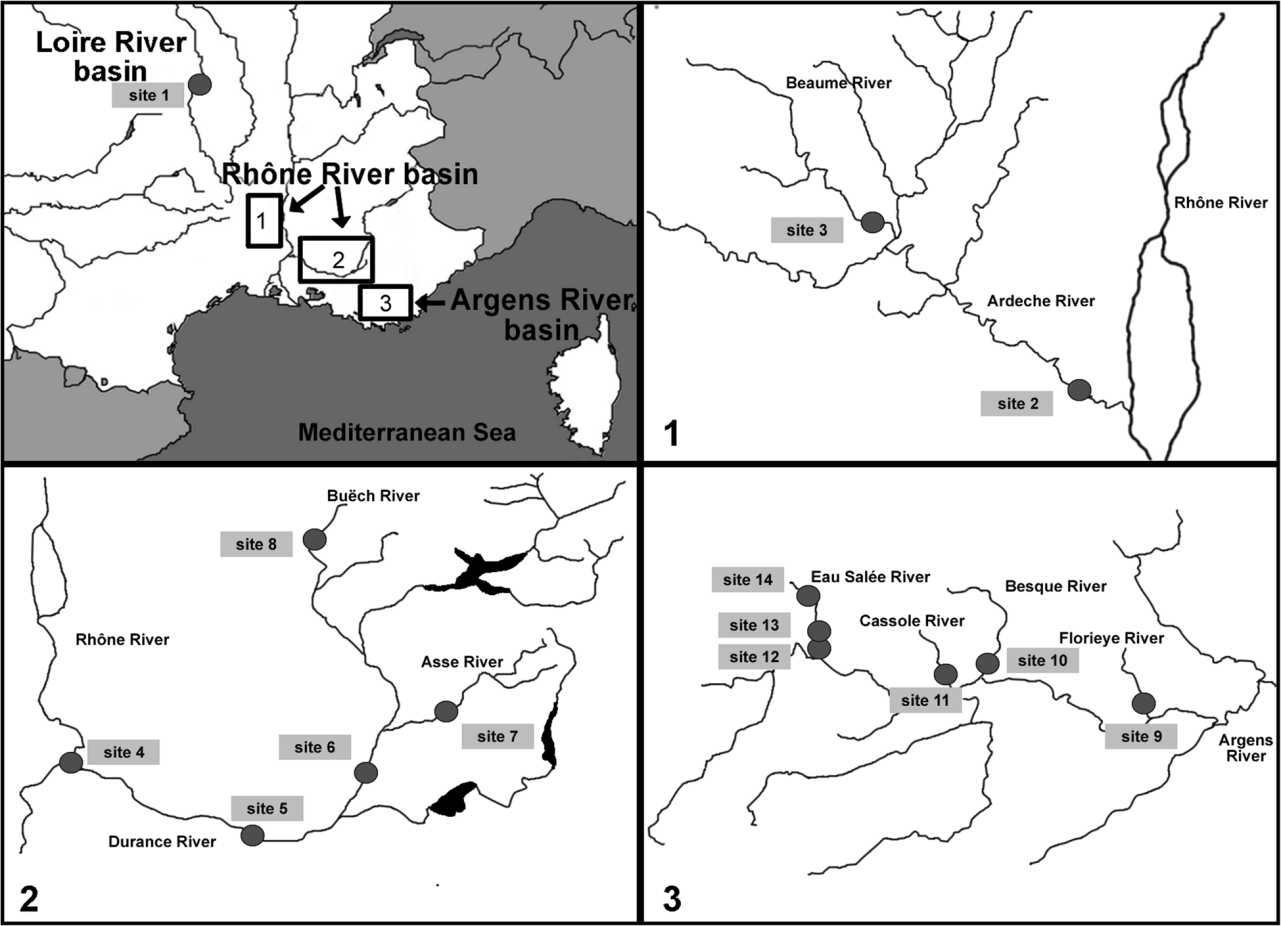
Maps of the study area and sampling localities (circles) of *B. meridionalis* and *B. barbus* populations studied

From 2007 to 2014, 349 *B. barbus* (BB), *B. meridionalis* (BM) and hybrid (H) individuals were collected in France from the allopatric BB and BM populations (site 1 on the Loire River basin and site 14 on the Argens River basin, respectively), the Rhône late sympatric populations (sites 2–3 on the River Ardèche and sites 4–8 on the River Durance of the Rhône River basin), and the Argens recent sympatric populations (sites 9–13 on the Argens River basin); (Fig. 1, Table 1). The full information on the site names and their coordinates are shown in Additional file 1: Table S1. To remove the effect of temporal variation, fish were sampled only in the summer period (i.e. July-August) when the highest parasite diversity and high abundance of many metazoan parasite species are expected. The water temperature was measured (in °C) in each locality. Fish were measured (standard body length in mm), transported to the laboratory, and subsequently examined for metazoan parasites.

**Table 1:** Characteristics of *Barbus* populations and their metazoan parasite communities. Mean values of total metazoan parasite abundance ± standard deviation (SD), bias-corrected Chao1 richness estimator ± SD, and the averaged prevalence ± SD for each parasite species of component populations for *B. barbus* (BB) and *B. meridionalis* (BM) populations and their respective hybrids (H). Population sample sizes (*n*) were assessed using the Introgress software package

Locality	Population	*n*	T^a^	Year	Abundance	Chao1	Averaged prevalence
Loire River							
Site 1	BB	21	22.7	2008; 2012	147.47 ± 151.26	12.23 ± 3.79	10.77 ± 23.78
Rhône River							
Site 2	BB	13	23	2012	302.92 ± 444.28	9.02 ± 1.91	11.94 ± 24.70
Site 3	BB	27	18	2010	143.74 ± 254.17	13.86 ± 3.01	12.57 ± 21.84
Site 4	BB	30	20.2	2011; 2012	184.50 ± 97.24	12.58 ± 2.09	15.53 ± 28.20
Site 5	BB	27	19.9	2007; 2010	193.44 ± 115.18	14.23 ± 2.67	16.67 ± 28.05
Site 6	BB	15	19.4	2010; 2011	148.67 ± 113.08	10.89 ± 2.81	14.39 ± 27.40
Site 7	BB	12	21.6	2010	148.50 ± 190.40	12.59 ± 2.42	15.35 ± 25.45
Site 8	BB	21	17.3	2010; 2011	164.67 ± 115.30	12.11 ± 2.80	14.66 ± 29.03
Argens River							
Site 9	BB	14	18.8	2013; 2014	17.86 ± 19.80	5.21 ± 1.15	8.08 ± 20.81
	BM	13			20.85 ± 11.90	6.72 ± 1.16	9.71 ± 21.43
	H	14			67.21 ± 122.70	8.58 ± 2.70	9.58 ± 21.29
Site 10	BB	2	18.7	2013	31.00 ± 21.21	4.27 ± 1.03	10.53 ± 26.40
	H	21			61.38 ± 125.08	8.79 ± 2.03	8.39 ± 18.96
Site 11	BM	29	17.5	2007; 2012; 2014	166.45 ± 311.53	9.79 ± 2.22	9.80 ± 22.41
	H	17			74.12 ± 117.09	10.12 ± 3.17	9.90 ± 21.10
Site 12	BB	9	17.3	2007	140.56 ± 119.36	8.36 ± 1.50	13.16 ± 27.44
	H	10			89.00 ± 95.82	9.39 ± 2.90	10.79 ± 21.23
Site 13	H	19	16.5	2013	26.89 ± 31.35	6.16 ± 1.40	6.65 ± 17.46
Site 14	BM	39	15.9	2013: 2014	356.08 ± 898.61	12.63 ± 1.82	12.89 ± 21.48

^a^T: mean water temperature in °C

### Microsatellite genotyping of fish individuals

Microsatellite markers developed for the *Barbus* species [34–36] were used to identify the fish species in our study. Genomic DNA was isolated from fin clip samples stored in 96% ethanol using DNeasy Blood & Tissue Kit (Qiagen GmbH, Hilden, Germany). Further diluted DNA (approx. 10 ng/l) served as a template in the following multiplex PCR analysis of microsatellite loci following the protocol described in [34]. Amplicons were analyzed on an ABI PRISM 3130 Genetic Analyzer (Applied Biosystems) using 500LIZ® Size Standard (Applied Biosystems) and Hi-Di™ Formamid (Applied Biosystems), and genotypes were finally scored using GeneMapper Software version 4.0 (Applied Biosystems). Due to the tetraploid genome of the investigated *Barbus* spp., only selected microsatellite loci with probable disomic inheritance [34] were applied in our study.

The MICRO-CHECKER program [37] was used to check for microsatellite null alleles in the *Barbus* populations and, subsequently, locus Barb21 was excluded from our study. Overall, 19 loci (Barbus2, Barbus22, Barbus26, Barbus28, Barbus31, Barbus32, Barbus36, Barbus37, Barbus40, Barbus41, Barbus47, Barbus49, Barbus50, Barbus56, Barbus62, Barbus63, Barbus65, Barb59 and Barb79) were used afterwards following a Bayesian clustering approach implemented in the STRUCTURE software [38]. The program was run for five independent runs assuming an admixture model and the model of correlated allele frequencies, using 1,000,000 iterations after a burn-in period of 100,000 iterations for K = 2 clusters. The *Introgress* package [39] implemented in the R statistical software was used to calculate the hybridization index (h-index) in sympatric populations. Allopatric populations of the Loire River and the Argens River basins (i.e. sites 1 and 14) were set in this software as parental BB and BM populations, respectively. First, the interspecific differentiation index (D) between allelic frequencies in parental populations was computed. Subsequently, 14 microsatellites with D ≥ 0.80 (Barbus22, Barbus26, Barbus31, Barbus32, Barbus37, Barbus47, Barbus49, Barbus50, Barbus56, Barbus62, Barbus65, Barbus63, Barb59 and Barb79) were selected for estimation of the h-index in sympatric populations, as applied in Andrés et al. [40]. In general, the resultant h-indices of zero and one should be used to determine pure individuals. Since the occurrence of BB in the Argens River basin is a result of introduction from the River Durance, we applied two approaches for BB designation (i) using an h-index of zero and (ii) using an h-index of up to 0.11. As similar results were obtained using both approaches (Additional file 2: Table S2; Additional file 3: Table S3; Additional file 4: Table S4), we presented only the results when the individuals with an h-index up to 0.11 were treated as BB and those with an h-index between 0.11 and 1 were treated as H individuals.

### Quantitative and qualitative comparisons of metazoan parasite communities

Fish dissection was performed following Ergens & Lom [41]. In our study, fins, gills, eyes, heart, kidney, spleen, hepathopancreas, intestine, gonads, gall-bladder, and swim bladder were examined for all metazoan parasites using a stereo microscope Olympus SZX7. Parasites were fixed as described in Lamková et al. [42] and, subsequently, identified using a light microscope (Olympus BX50) equipped with phase-contrast, differential interference contrast, and Olympus Stream Motion 1.9.2 digital image analysis software. Parasites were identified using the available identification keys and publications providing keys to the identification of metazoan parasites, e.g. [43–46]. Measures of parasite infection, i.e. prevalence and abundance of metazoan parasites were calculated according to Bush et al. [47]. The individual abundance of the myxozoan parasites was not taken into consideration (because these parasites cannot be quantified as in the case of other metazoan parasites) and only the prevalence of this parasitic group was taken into account in the analyses. The effect of sampling effort on parasite species richness was corrected using the Chao1 estimator [48], and was calculated using the EstimateS program [49] on the basis of abundance data excluding data on myxozoan parasites. Similarities between metazoan parasite communities based on presence/absence data (Jaccard index) were computed in PAST [50].

### Statistical analyses

Spearman’s rank correlation was computed between individual admixture q-values and h-index obtained by STRUCTURE software and Introgress package software, respectively. Metazoan parasite abundance and averaged prevalence were log-transformed prior to statistical analyses. Kolmogorov-Smirnov test was used for normality data assessment. Subsequently, Bonferroni *post-hoc* tests following General Linear Model (GLM) were used to compare the estimated marginal means of total abundance, prevalence and species richness adjusted for fish body length, water temperature, and sampling years between BB, BM and H from different river basins. Since all fish individuals were infected with at least one parasitic species (i.e. the overall prevalence was 100% in each fish population), the average of prevalences for each parasite species across the fish populations was used in our study (further referred to as averaged prevalence).

For the River Argens, GLM analyses were conducted to investigate the potential effects of different factors, i.e. host (BB, BM or H), locality (site), sampling year, water temperature, and host body length on the abundance, averaged prevalence, and species richness of metazoan parasites found in *Barbus* individuals.

## Results

### Genetic composition of *Barbus* spp. populations

**Fig. 2:**
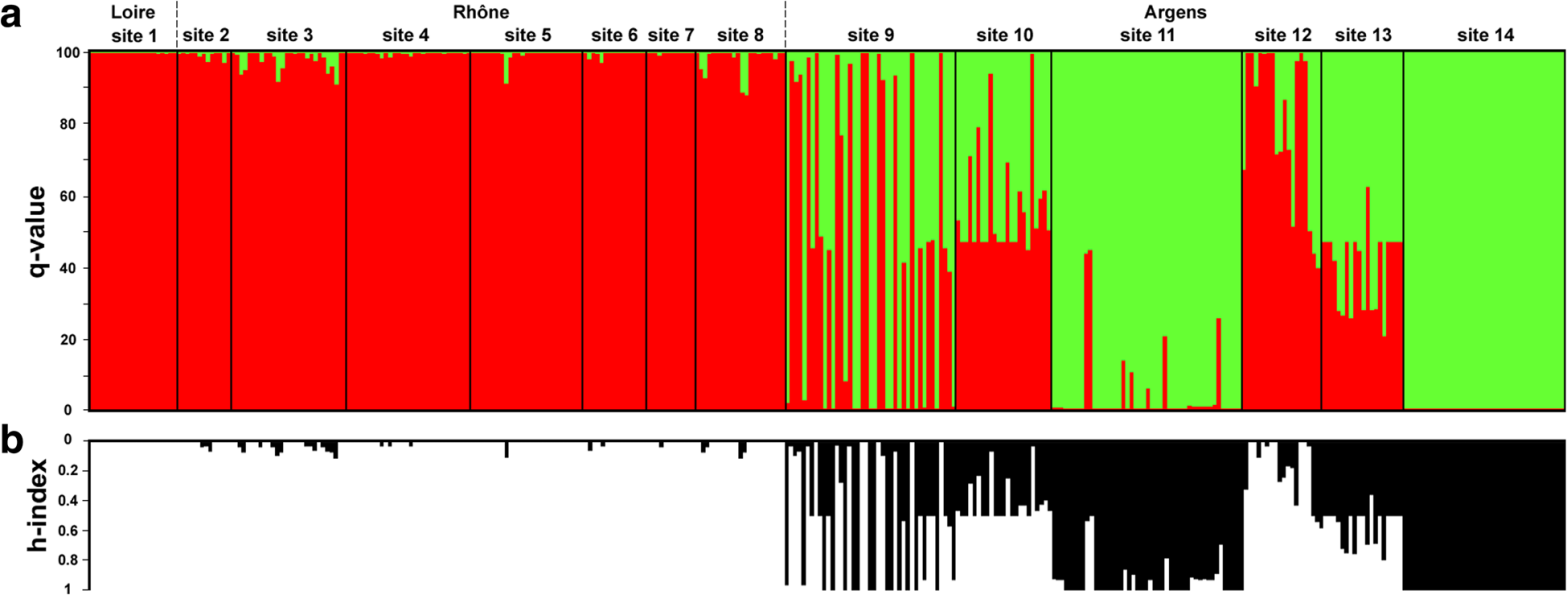
Genetic structure of *B. meridionalis* and *B. barbus* populations inferred from (**a**) STRUCTURE analysis showing probabilities of individuals’ assignment to *B. barbus* (*red*) and *B. meridionalis* (*green*), and (**b**) the Introgress software package displaying bars’ lengths proportional to the probability that individuals belong to *B. barbus*. Each vertical bar represents one individual

Based on microsatellite markers, STRUCTURE analysis confirmed the existence of one allopatric BB population in the Loire River basin (Site 1) and one allopatric BM population in the Argens River basin (Site 14). A low level of admixture between the populations of two *Barbus* species was revealed in the Rhône River basin (q < 0.12). In contrast, the extent of population admixture in the Argens River basin was high except for the abovementioned the Argens allopatric BM population (Fig. 2). The values of h-index obtained by the Introgress software package resembled the overall picture of the individual admixture obtained by STRUCTURE (Fig. 2) and correlated significantly with the q-values (Spearman’s rank correlation, *r*_(373)_ = 0.97, *P* < 0.001). An h-index up to 0.11 was revealed in populations of the Rhône River basin. Using this value as an upper limit for *B. barbus* individuals, 25 *B. barbus*, 42 *B. meridionalis* and 81 hybrids were detected within the Argens River basin (i.e. a system with a very recent introduction of *B. barbus*), where we considered *Barbus* populations with the occurrence of hybrids as sympatric populations. In the Argens River basin, the co-existence of both pure species was documented only in Site 9. The *Barbus* sample originating from Site 13 was composed entirely of hybrid individuals (Table 1, Fig. 2).

### Composition of metazoan parasite communities in *Barbus *spp. populations from different river basins

**Fig. 3:**
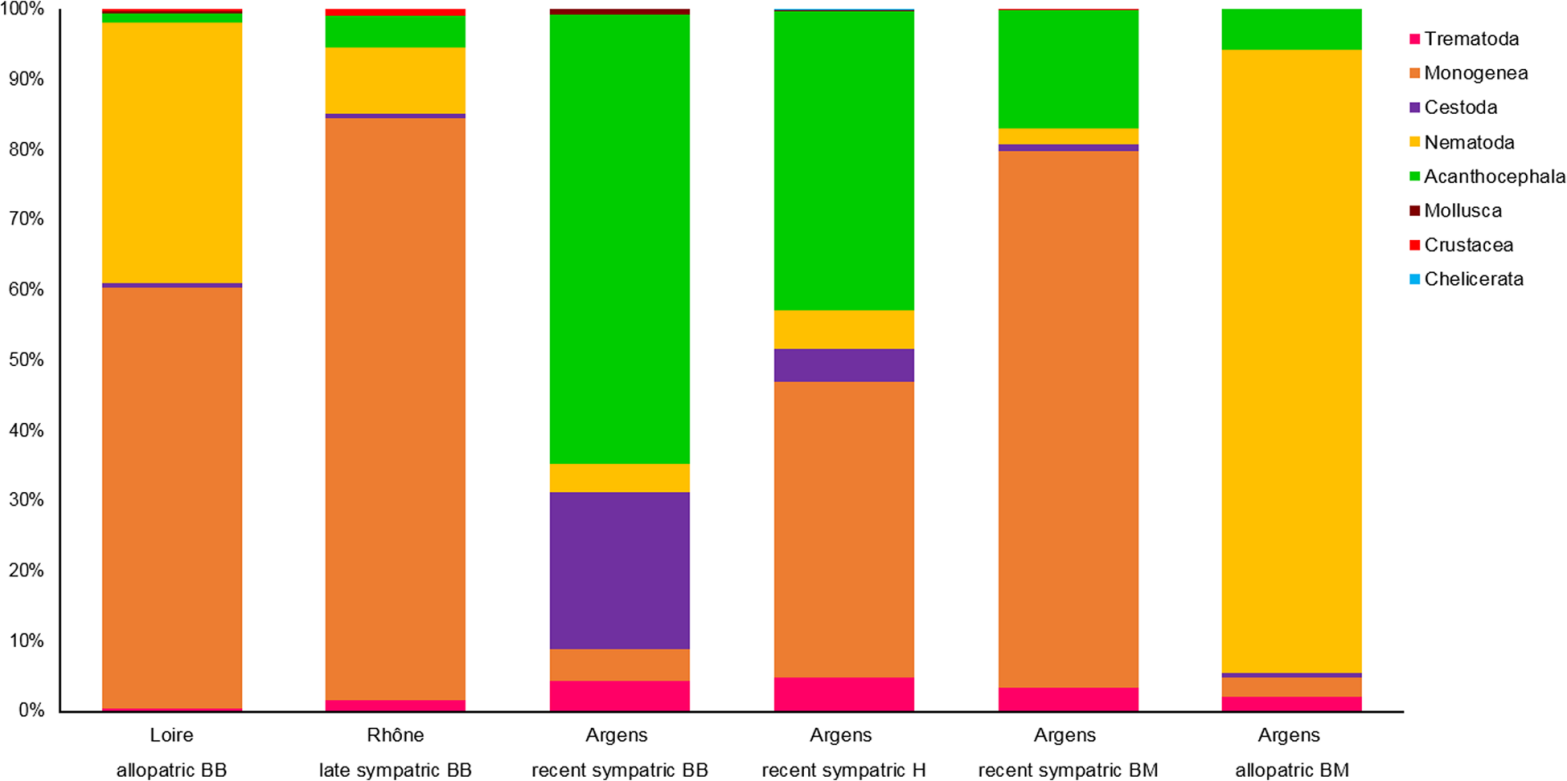
Metazoan parasites in *Barbus* spp. populations. Proportions of parasite groups in metazoan parasite communities in the populations of *B. barbus* (BB), *B. meridionalis* (BM) and their hybrids (H) from the three river basins

Examination of the studied *Barbus* spp. populations for metazoan parasites revealed the presence of parasites belonging to different parasitic groups (Myxozoa, Trematoda, Monogenea, Cestoda, Nematoda, Acanthocephala, Mollusca, Chelicerata and Crustacea). The prevalence and mean abundance of each metazoan parasite species are listed in Table 2. Detailed information on the composition of parasite communities per site is shown in Additional file 5: Table S5. Monogeneans (especially *Dactylogyrus* spp.) were the most dominant groups of parasites in the Loire allopatric BB and the Rhône late sympatric BB, while they were rare in the Argens recent sympatric BB. Above all, monogeneans and acanthocephalans (specifically *Gyrodactylus* spp. and *Pomphorhynchus tereticollis*, respectively) were dominant in the Argens recent sympatric BM and H. In a single Argens allopatric BM population, nematodes represented the most dominant and abundant parasite group (Table 2, Fig. 3).

**Table 2:** Metazoan parasite communities in *Barbus* spp. populations. Mean abundance (A) ± standard deviation (SD) and prevalence (P, in %) of metazoan parasites in *B. barbus* (BB), *B. meridionalis* (BM) and their hybrids (H)

	Loire allopatric BB		Rhône late sympatric BB		Argens recent sympatric BB		Argens recent sympatric H		Argens recent sympatric BM		Argens allopatric BM	
	(*n* = 21)		(*n* = 145)		(*n* = 25)		(*n* = 81)		(*n* = 42)		(*n* = 39)	
	A ± SD	P	A ± SD	P	A ± SD	P	A ± SD	P	A ± SD	P	A ± SD	P
Myxozoa												
*Myxobolus* spp.	–	38	–	66	–	60	–	28	–	21	–	21
Trematoda												
*Allocreadium isoporum*	0.14 ± 0.36	14	–	–	–	–	2.27 ± 8.25	20	2.57 ± 7.80	31	7.13 ± 22.19	49
*Apharyngostrigea* sp.	–	–	0.26 ± 3.07	1	0.28 ± 0.89	12	0.16 ± 1.44	1	1.40 ± 9.10	2	–	–
*Clinostomum complanatum*	–	–	0.39 ± 2.77	12	–	–	–	–	–	–	–	–
*Diplostomum* spp.	0.14 ± 0.65	5	1.87 ± 6.29	38	0.80 ± 1.53	28	0.12 ± 0.53	7	–	–	–	–
Echinostomatidae gen. sp.	0.24 ±1.09	5	–	–	–	–	–	–	–	–	–	–
Digenea fam. gen. spp.	0.05 ± 0.22	5	0.19 ± 2.16	1	–	–	–	–	–	–	–	–
*Holostephanus* sp.	–	–	–	–	1.68 ± 4.13	24	0.35 ± 1.69	5	–	–	–	–
*Tylodelphys* sp.	–	–	0.10 ± 0.53	5	–	–	0.02 ± 0.22	1	–	–	–	–
Monogenea												
*Dactylogyrus* sp.	–	–	–	–	–	–	–	–	–	–	0.03 ± 0.16	3
*Dactylogyrus extensus*	–	–	–	–	–	–	0.07 ± 0.38	5	0.05 ± 0.22	5	–	–
*Dactylogyrus carpathicus*	3.19 ± 14.39	10	57.67 ± 76.65	79	–	–	–	–	–	–	–	–
*Dactylogyrus malleus*	81.95 ± 88.38	100	70.14 ± 160.06	90	0.60 ± 2.40	16	0.16 ± 0.70	7	–	–	–	–
*Gyrodactylus hemibarbi*	–	–	0.34 ± 0.97	17	0.12 ± 0.43	8	0.63 ± 1.50	20	4.40 ± 6.49	74	2.49 ± 4.23	56
* Gyrodactylus katharineri*	–	–	0.06 ± 0.27	6	0.92 ± 1.63	40	0.67 ± 1.10	35	0.52 ± 1.37	24	–	–
*Gyrodactylus markewitschi*	3.48 ±10.53	33	20.59 ± 87.32	34	1.12 ± 5.40	8	17.96 ± 80.32	25	6.10 ± 18.82	43	2.82 ± 7.81	41
*Gyrodactylus sprostonae*	–	–	–	–	–	–	5.91 ± 50.59	2	81.79 ± 261.36	26	4.00 ± 12.18	26
*Paradiplozoon homoion*	–	–	0.23 ± 1.02	12	0.04 ± 0.02	4	0.04 ± 0.19	4	–	–	0.38 ±0.81	23
Cestoda												
*Bathybothrium rectangulum*	0.90 ± 3.06	19	1.08 ± 3.64	16	6.6 ± 15.52	32	2.02 ± 9.15	19	1.17 ± 2.25	21	0.74 ± 1.27	36
*Schyzocotyle acheilognathi*	–	–	0.06 ± 0.37	3	–	–	–	–	–	–	–	–
*Caryophyllaeus brachycollis*	–	–	0.01 ± 0.12	1	7.48 ± 22.63	28	0.75 ± 3.43	12	0.05 ± 0.31	2	1.85 ± 6.11	28
*Proteocephalus torulosus*	–	–	–	–	–	–	–	–	–	–	0.15 ± 0.59	8
Nematoda												
*Contracaecum* sp.	–	–	0.30 ± 2.15	8	–	–	–	–	–	–	–	–
*Pseudocapillaria tomentosa*	1.00 ± 4.15	10	0.47 ± 2.02	10	0.08 ± 0.28	8	0.75 ± 2.37	21	0.64 ± 2.09	14	0.31 ± 0.61	23
*Rhabdochona hellichi*	53.90 ± 67.03	100	14.99 ± 22.15	72	2.56 ± 4.55	48	2.63 ± 9.00	30	2.14 ± 6.33	19	2.23 ±5.72	41
Nematoda fam. gen. sp. 1	–	–	–	–	–	–	–	–	0.02 ± 0.15	2	0.28 ± 1.32	8
Nematoda fam. gen. sp. 2	–	–	1.01 ± 12.04	1	–	–	–	–	–	–	–	–
Nematoda fam. gen. sp. 3	–	–	–	–	–	–	–	–	–	–	313.05 ± 901.63	33
Acanthocephala												
*Acanthocephalus anguillae*	0.05 ± 0.22	5	0.25 ±2.04	4	–	–	–	–	–	–	–	–
Acanthocephala fam. gen spp.	–	–	0.03 ± 0.26	1	–	–	0.15 ± 1.33	1	–	–	–	–
*Pomphorhynchus tereticollis*	1.67 ± 4.86	19	7.97 ± 23.56	39	40.36 ± 73.65	44	25.59 ± 39.91	80	20.50 ± 19.95	83	20.62 ± 17.64	95
Mollusca												
*Anodonta* spp.	0.52 ± 0.68	43	0.15 ± 0.69	6	0.44 ± 0.96	24	0.10 ± 0.46	5	–	–	–	–
Crustacea												
*Argulus coregoni*	–	–	0.01 ± 0.08	1	–	–	–	–	–	–	–	–
*Ergasilus sieboldi*	0.43 ±1.96	5	1.32 ± 5.06	21	–	–	–	–	0.02 ± 0.15	2	–	–
*Tracheliastes polycolpus*	–	–	0.17 ± 0.68	10	–	–	–	–	–	–	–	–
Chelicerata												
*Hydrozetes* sp.	–	–	–	–	–	–	0.01 ± 0.11	1	–	–	–	–

### Similarity in metazoan parasite communities between *Barbus* spp. populations

High Jaccard index value was found between the Argens recent sympatric BB and H individuals (0.71), while lower similarity in parasite communities (0.57) was found between the Argens recent sympatric BM and H. Parasite communities in the Argens sympatric BB were more similar to parasite communities in the Rhône late sympatric BB (0.52) than to those in the Loire allopatric BB (0.43). The parasite communities in the Argens recent sympatric BB and the Argens recent sympatric BM were more similar (0.50) than those in the Argens recent sympatric BB and the Argens allopatric BM (0.43; Table 3).

**Table 3:** Jaccard similarity indices for metazoan parasite communities in *Barbus* spp. populations

	Loire allopatric BB	Rhône late sympatric BB	Argens recent sympatric BB	Argens recent sympatric H	Argens recent sympatric BM	Argens allopatric BM
Loire allopatric BB	–					
Rhône late sympatric BB	0.46	–				
Argens recent sympatric BB	0.43	0.52	–			
Argens recent sympatric H	0.38	0.52	0.71	–		
Argens recent sympatric BM	0.36	0.37	0.50	0.57	–	
Argens allopatric BM	0.30	0.28	0.43	0.44	0.58	–

*Abbreviations*: BB, *B. barbus*; BM, *B. meridionalis*; *H*, hybrids

### Total abundance, averaged prevalence and species richness of metazoan parasites in *Barbus* spp. populations

**Fig. 4:**
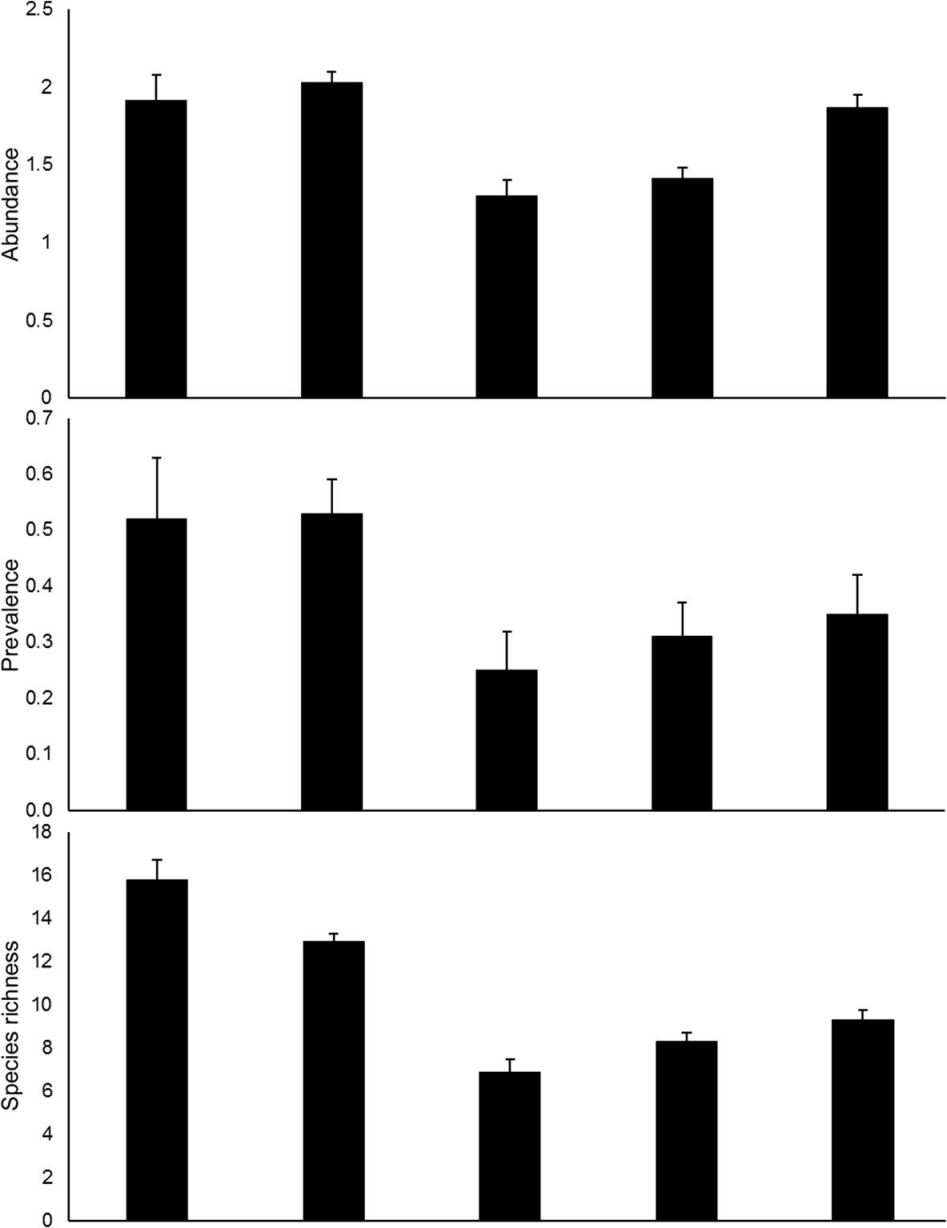
Metazoan parasite abundance, prevalence and species richness in *Barbus* spp. populations. Mean values (+ standard errors) of log-transformed total abundance and averaged prevalence, and species richness (Chao1 index) of metazoan parasites in *B. barbus* (BB), *B. meridionalis* (BM) and their hybrids (H) corrected for fish body length, water temperature and sampling year

Significant differences in abundance, averaged prevalence, and species richness (GLM, abundance: whole model *F*_(4,340)_ = 18.05, *P* < 0.001; averaged prevalence: whole model *F*_(4,1241)_ = 1.70, *P* = 0.029; species richness: whole model *F*_(4,340)_ = 23.24, *P* < 0.001) of metazoan parasites between *Barbus* groups were revealed (see Additional file 3: Table S3 and Additional file 4: Table S4 for detailed statistics). After controlling for the covariates, Bonferroni *post-hoc* tests revealed no significant differences in metazoan parasite abundance and averaged prevalence between the Loire allopatric BB and the Rhône late sympatric BB (*P* > 0.05). However, significantly lower species richness was revealed in the Rhône late sympatric BB than in the Loire allopatric BB (*P* = 0.008). Significantly lower values of abundance and species richness (*P* < 0.001), and lower but not significantly different averaged prevalence (*P* > 0.05) of metazoan parasites were found in the Argens recent sympatric BB in comparison with the Rhône late sympatric BB. In the Argens River basin, significantly lower metazoan abundance (*P* < 0.001) and species richness (*P* = 0.004), and lower but not significantly different averaged prevalence (*P* > 0.05) were found in BB when compared to BM. Metazoan parasite abundance, averaged prevalence, and species richness in H tended to be intermediate between pure species of the Argens River basin. However, statistically significant difference was only revealed in the case of metazoan parasite abundance between H and BM (*P* < 0.001); (Fig. 4, Additional file 4: Table S4).

### Factors influencing parasitism in the *B. barbus* × *B. meridionalis* recent hybrid zone

In the Argens River basin, significant effects of locality (*F*_(5,174)_ = 11.42, *P* < 0.001), sampling year (*F*_(3,174)_ = 7.99, *P* < 0.001), water temperature (*F*_(1,174)_ = 6.33, *P* = 0.013), and fish length (*F*_(1,174)_ = 20.15, *P* < 0.001) on metazoan parasite abundance were revealed, while effect of host was not significant in GLM (whole model *R* ^2^ = 0.45, *F*_(12,174)_ = 11.55, *P* < 0.001). Host (*F*_(2,174)_ = 3.85, *P* = 0.012), locality (*F*_(5,124)_ = 20.87, *P* < 0.001), sampling year (*F*_(3,174)_ = 20.22, *P* < 0.001), water temperature (*F*_(1,174)_ =5.32, *P* = 0.030), and fish length (*F*_(1,174)_ = 4.16, *P* = 0.020) significantly affected the species richness of metazoan parasites (whole model *R* ^2^ = 0.74, *F*_(12,174)_ = 32.88, *P* < 0.001). Neither host, locality, sampling year, water temperature, nor host body length effected significantly averaged prevalence of metazoan parasites (whole model *R* ^2^ = 0.02, *F*_(12,709)_ = 1.01, *P* = 0.442).

## Discussion

This study explored composition of metazoan parasite communities in *Barbus* spp. populations of three river basins. No significant differences in the intensity of metazoan parasite infection was revealed between the Rhône late sympatric BB and the Loire allopatric BB. At the same time, lower number of parasitic taxa was revealed in the Rhône late sympatric BB than in the Loire allopatric BB. Low introgression of BM revealed by microsatellite markers in the Rhône late sympatric BB, therefore, does not provide a disadvantage in terms of high levels of parasite infection or high species richness in contrast to the Loire allopatric BB. Yet, a degree to which parasites have a negative impact on host vigour and fitness components is dependent on a particular parasite species, parasite genotype, or co-infection with other parasite species [51, 52]. This is, however, beyond the scope of our study. Several parasite species were shared between the Loire allopatric BB, the Rhône late sympatric BB, and the Argens recent sympatric BB, which indicates that these parasite species followed the expansion of BB. On the other hand, we detected considerably lower diversity and intensity of infection of metazoan parasites in the Argens recent sympatric BB in contrast to the Rhône late sympatric BB. Still, all parasite species except for *Holostephanus* sp. found in our study in the Argens recent sympatric BB were also present in the Rhône late sympatric BB (Table 2). These findings support the view that BB of the Argens River basin originates from the Durance River system. However, parasite species infecting the Argens recent sympatric BB represented only a small proportion of the metazoan parasite fauna found in the Rhône late sympatric BB (Table 2). The enemy release hypothesis suggests that individuals introduced outside their natural ranges may benefit from enemy release, e.g. predators or pathogens [53–55]. Our results concerning the significantly lower levels of parasite infection and lower number of metazoan parasites in the Argens recent sympatric BB when compared to the Rhône late sympatric BB are, therefore, in congruence with the general scenario of parasite loss after host introduction into a new environments [18]. Kennedy & Bush [56] revealed that the parasite communities of native *Onchorhynchus mykiss* populations were dominated by specialist helminth parasites, while the number of specialist helminths declined with the increasing distance of translocated host populations from their original heartland. In fish, monogeneans are considered to be the most host-specific parasites [57]. In our study, five of six monogenean species documented in the Rhône late sympatric BB were found also in the Argens recent sympatric BB. However, the absence of *Dactylogyrus carpathicus*, a highly abundant parasite species of the Rhône late sympatric BB, and the decrease in the abundance and prevalence of *Dactylogyrus malleus* in the Argens sympatric BB resulted in a shift in parasite communities from the dominance of monogenean parasites to a higher proportion of endoparasitic groups in total parasite numbers. Ondračková et al. [22] showed that the reduction in parasite numbers is dependent on the time after a colonization event and, therefore, a very late arrival of *B. barbus* into the Argens River tributaries can be expected. The first events involving the migration of BB from the River Durance to the waters of the Argens River system probably occurred in the period 1980–1990 [32]. Since an upstream migration range of up to several dozens of kilometres has been documented for BB individuals [58], colonization of the Argens River tributaries might thus have taken place at the turn of this century or even more recently.

Parasites often exhibit a shorter generation time, larger population size, and higher migration and mutation rates than their hosts. Consequently, as a result of co-evolutionary host-parasite interactions, local parasite adaptation, i.e. the better performance of parasites in their local hosts than in foreign ones, is expected [59]. Individuals introduced into novel areas may, therefore, benefit from the higher ability of parasites to adapt to their local hosts. By contrast, lower ability of parasites to infect their local hosts than alien ones or no differences in degrees of host resistance/susceptibility to parasites between local and non-indigenous hosts (i.e. no local adaptation), were previously documented [60, 61]. In our study, with respect to the Argens River basin, the lower parasite diversity and lower parameters of metazoan parasite infection found in introduced BB when compared to the local BM (Fig. 4) indicate that parasites are adapted to their local BM populations. On the other hand, elevated metazoan parasite infection in native BM might be a result of parasite transmission from introduced BB. Out of all the parasite species that infected both the Rhône late sympatric BB and the Argens recent sympatric BB individuals, and might, therefore, have been carried along with host introductions, almost 70% were also found in BM (Table 2). To our knowledge, and with the exception of this study, no investigation of metazoan parasite communities in fish from the Argens River system has so far been undertaken. We, therefore, cannot exclude the possibility that the parasite species that we reported from both BB and BM were already present in the Argens River basin before the introduction of BB. *Myxobolus* spp. parasites as well as endoparasites such as *Pseudocapillaria tomentosa*, *Caryophyllaeus brachycollis* and *Pomphorhynchus terreticollis*, or parasites belonging to the genus *Apharyngostrigea*, infect a wide range of fish hosts beside barbels [62–65]. On the other hand, *Bathybothrium rectangulum* and *Rhabdochona hellichi*, which are commonly found in barbels and rarely found in other fish [57], were already reported from both BB and BM [62, 65]. Similarly, *Gyrodactylus katharineri*, *G. hemibarbi* and *G. markewitchi* found in both *Barbus* species in our study, have already been documented from other cyprinids besides barbels, such as *Cyprinus carpio*, *Alburnus alburnus*, *Gobio gobio*, *Leuciscus cephalus* and *Gymnocephalus cornua* [66–68]; these cyprinids were already reported from the Argens River basin [33]. Finally, in our study, the monogenean parasite *Paradiplozoon homoion* occurred in very low abundance and prevalence on both BB and BM*.* Le Brun et al. [69] documented that *Paradiplozoon gracile* never parasitized BB, while it is present on BM and H individuals of these two species (*P. gracile* was proposed as a synonym of *P. homoion* [70]). Slightly higher values of *P. homoion* infection in BM than in BB of the Argens River basin and its absence in the Loire allopatric BB population may even indicate the opposite direction of transmission, i.e. from BM to BB. However, this parasite species was already reported in BB and exhibits a wide range of cyprinid hosts [43, 62, 63, 70]. Even if all these parasites that were, in our study, shared between barbel hosts were indeed present in the Argens River basin, contact zones between these two *Barbus* species may act as a reservoir for the reinforcement of infection of local BM from introduced BB. On the other hand, the exposure of susceptible non-native host species to native parasites might lead to parasite spillback, i.e. an increase in the impact of a disease on a local host *via* transmission from a competent alien host [71]. This may be supported by higher similarity in parasite communities between the Argens recent sympatric BB and the Argens recent sympatric BM than between the Argens recent sympatric BB and the Argens allopatric BM. In general, similar parasite communities are expected in hosts living in sympatric areas as a result of host contact, which facilitates parasite transmission [72]. Our findings, therefore, highlight the risk of possible parasite transmission from invasive BB to endemic BM. Šimková et al. [72] indicated that local and endemic *Parachondrostoma toxostoma* became infected by *Dactylogyrus* parasites after contact with the invasive *Chondrostoma nasus* in southern France. In our study, *Dactylogyrus* parasites were very rare in BM. *Dactylogyrus malleus*, the only representative of the genus which followed the expansion of BB, was not transmitted to BM, even though H individuals are susceptible to infection with this species (Table 2). The higher infection levels of these parasites, as was found in the Loire allopatric BB and the Rhône late sympatric BB, and subsequently their capacity to be transmitted to local BM might, therefore, be expected in the very near future.

A continuum of hybrid genotypes and phenotypes between parental species may fill the gap between the two hybridizing species and facilitate parasite transmission [26]. As a result, we might expect that mixed genotypes arising by introgressive hybridization should enable more opportunities for infection by parasite species which naturally track parental species, i.e. hybrids will be infected by parasites of both parental taxa [24, 25]. In our study, hybrids of the Argens River basin were, overall, parasitized by a number of parasite species intermediate between pure species (Fig. 4). However, in localities where at least one parental species was present, hybrid individuals tended to be parasitized by more parasite species (Tables 1 and 2). Moreover, the effect of host on the number of parasite species which infected *Barbus* populations was confirmed by the results of GLM analysis and may be a result of differences in host ethology and ecological preference [69]. Thus, our findings suggest that hybrids represent “bridges” for parasite infection between invasive and endemic species. From this point of view, it seems that BB represents a potential threat to local BM in terms of the transmission or increased impact of metazoan parasites on local BM *via* hybridization. In fact, all parasites that infected both parental taxa were also detected in hybrids of the Argens River basin. Consequently, we may conclude that metazoan parasites represent important biomarkers of BB and BM hybridization in the Argens River basin, as was also reported for *Dactylogyrus* parasites in *Alburnus alburnus* × *Rutilus rutilus* hybrids (Lake Mikri Prespa, Greece; [24]). Šimková et al*.* [25] reported that *Cyprinus carpio* × *Carassius gibelio* hybrids were also parasitized by a greater variety of parasite species than pure hosts; however, they remained less susceptible to metazoan parasites, which could be a result of so-called hybrid vigour [73]. In our study, hybrids displayed higher fitness overall than BM in terms of lower parasite abundance and resembled BB more in this respect (Fig. 4). Slightly greater similarity in parasite communities was also revealed between H and invasive BB than between H and endemic BM, based on presence/absence data. Phillipart & Berrebi [74] showed that experimental crossing between female BB and male BM resulted in F1 hybrids with a similar size structure to BB and favored female F1 hybrids. As a result, the maternal effect may influence offspring (in our case, hybrid) susceptibility to parasites [75]. Yet, hybrid individuals displayed different levels of susceptibility to metazoan parasites (i.e. higher or lower abundance), in contrast to the parental taxa in localities where parental species were also present (Table 1). These findings are consistent with the results of GLM, which showed no effect of host on metazoan parasite abundance, while significant effects of host body size, locality, sampling year, and water temperature on metazoan parasite abundance were demonstrated. Since parasite infection in hosts is unstable in the time and space driven by host-parasite interactions and environmental forces [76–78], our findings indicate that both individual host characteristics (i.e. host genotype) and environmental factors together significantly influence spatio-temporal distribution of metazoan parasite communities in the BB × BM hybrid system. Since metazoan parasites with the complex life-cycle constitute a substantial fraction of metazoan parasite communities in *Barbus* spp. of the Argens River basin, spatial differences in diversity of parasite communities found in our study might be also partially shaped by the availability and abundance of intermediate hosts, i.e. the presence of Nematode sp. 3 in the Argens allopatric BM. The effect of intermediate host abundance on the parasite diversity was previously shown in eels [79].

## Conclusions

On the basis of our results, we may conclude that metazoan parasites extend along the expansion range of invasive *B. barbus*. While similar levels of metazoan parasite infection were revealed in *B. barbus* of the Loire River basin (i.e. in the absence of *B. meridionalis*), and *B. barbus* of the Rhône River basin (i.e. where there is a low level of microsatellite introgression from *B. meridionalis*), *B. barbus* recently introduced into the Argens River was shown to profit from enemy release after its arrival from the River Durance by displaying lower susceptibility to metazoan parasites in contrast to the source populations. Concerning the Argens River basin, lower levels of parasite infection in populations of *B. barbus* in comparison to *B. meridionalis* and a similarity in metazoan parasite communities with those found in the Rhône River basin support the idea that the introduction of *B. barbus* into the watersheds of the River Argens from the River Durance is of very recent origin. The infection of hybrids by metazoan parasites found in both parental species in the River Argens supports the existence of hybridization between *B. barbus* and *B. meridionalis* and indicates that parasites along with molecular markers may be used as powerful tools for detecting recent hybridization events. The transmission of parasites *via* introgressive hybridization and higher parasite infection in *B. barbus* from the Loire River basin and the Rhône River basin indicated in our study may highlight the potential risk of non-native *B. barbus* having an increased disease impact on endangered *B. meridionalis*.

## Additional files

Additional file 1: Table S1. Site names and their geographical coordinates. (XLSX 10 kb)
Additional file 2: Table S2. Metazoan parasite abundance, prevalence and species richness in *Barbus* spp. populations. (XLSX 11 kb)
Additional file 3: Table S3. Results of ANCOVA tests. (XLSX 16 kb)
Additional file 4: Table S4.
*P*-values of Bonferroni *post-hoc* tests. (XLSX 13 kb)
Additional file 5: Table S5. Metazoan parasite communities in *Barbus* spp. populations. (XLSX 21 kb)

## Abbreviations

BB: *Barbus barbus*
BM: *Barbus meridionalis*
H: Hybrids
GLM: General linear model
