# Erratum to: Metazoan parasite communities: support for the biological invasion of *Barbus barbus* and its hybridization with the endemic *Barbus meridionalis*

**DOI:** 10.1186/s13071-017-2214-5

**Published:** 2017-05-30

**Authors:** Lenka Gettová, André Gilles, Andrea Šimková

**Affiliations:** 1 Department of Botany and Zoology, Faculty of Science, Masaryk University, Kotlářská 2, 61137 Brno, Czech Republic; 2 Aix-Marseille Université, IMBE, UMR CNRS 7263, Evolution Génome Environnement, Case 36, 3 Place Victor Hugo, 13331 Marseille Cedex 3, France

## Erratum

After the publication of the article [1], it was realised that X-axis labels are missing in Fig. 4. The corrected Fig. 4 is included below.

**Fig. 4:**
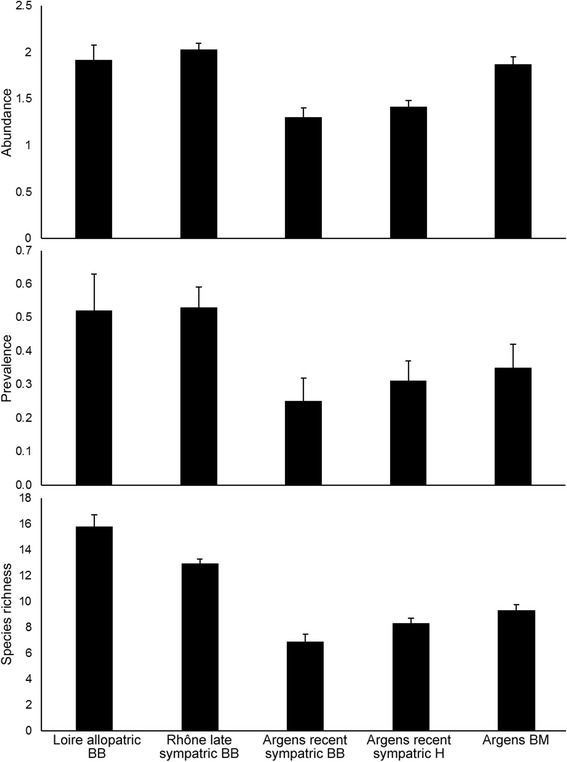
Metazoan parasite abundance, prevalence and species richness in *Barbus* spp. populations. Mean values (+ standard errors) of log-transformed total abundance and averaged prevalence, and species richness (Chao1 index) of metazoan parasites in *B. barbus* (BB), *B. meridionalis* (BM) and their hybrids (H) corrected for fish body length, water temperature and sampling year
